# Monotherapy with Biologics for Generalized Pustular Psoriasis: A Systematic Review of Comparative Interventional Studies with an Exploratory Network Meta-Analysis

**DOI:** 10.3390/medsci14020307

**Published:** 2026-06-11

**Authors:** Aditya K. Gupta, Mary A. Bamimore, Tong Wang, Mesbah Talukder, Vincent Piguet

**Affiliations:** 1 Division of Dermatology, Temerty Faculty of Medicine, University of Toronto, Toronto, ON M5S 1A8, Canada; vincent.piguet@utoronto.ca; 2 Mediprobe Research Inc., London, ON N5X 2P1, Canada; mbamimor@alumni.uwo.ca (M.A.B.); twang@mediproberesearch.com (T.W.); mesbah.talukder@bracu.ac.bd (M.T.); 3 School of Pharmacy, BRAC University, Dhaka 1212, Bangladesh; 4 Division of Dermatology, Women’s College Hospital, Toronto, ON M5G 1B2, Canada

**Keywords:** generalized pustular psoriasis, network-meta analysis, pustulation, biologics

## Abstract

**Background**—Generalized pustular psoriasis (GPP) is a rare and severe inflammatory skin disorder—and evidence regarding relative impact of treatments thereof is currently scant. **Objective**—We aimed to systematically review and narratively synthesize comparative interventional therapies for GPP and secondarily explore their relative effectiveness through exploratory network meta-analyses (NMAs). **Methods**—Comprehensive searches were performed in PubMed and EMBASE to identify comparative interventional studies that investigated the impact of biologics in GPP. Bayesian NMAs were conducted only for exploratory analyses. **Results**—Eleven studies met our inclusion criteria and data from 4 of the 11 were used for NMAs. Methodological heterogeneity was evident; the various biologics demonstrated effectiveness in treating GPP. Inhibitors of interleukin (IL)-36 (e.g., spesolimab) resulted in rapid pustular clearance within one week and sustained reductions in flare occurrence. Inhibitors targeting IL-17, IL-23, TNF, and IL-12/23 also demonstrated high response rates, durable disease control, and improvements in quality of life among diverse patient populations. Results from our exploratory NMAs revealed patterns of relative effectiveness with IL-17 and IL-36 inhibitors that are consistent with the existing literature. However, methodological limitations across the four studies deterred us from making conclusive inferences. **Conclusions**—Biologic therapies provide significant clinical benefit in patients with GPP. Our narrative syntheses highlight the need for future quantitative syntheses.

## 1. Introduction

Generalized pustular psoriasis (GPP), a rare form of psoriasis, is a life-threatening inflammatory condition mainly characterized by diffuse eruptions of sterile pustules [1,2]. The condition is linked to various genetic mutations that compromise integrity of the epidermal barrier and disrupt innate immune signaling. Generalized pustular psoriasis can be triggered by abrupt corticosteroid withdrawal, infections, medications, and physiological stress. Risk factors include metabolic syndrome, polyarthritis, and a family history of psoriasis [3]. Generalized pustular psoriasis is characterized by neutrophilic skin infiltration causing pustules and systemic inflammation. Diagnosis is based on clinical findings, histopathology, laboratory tests, and exclusion of infection [4]. Management often necessitates hospitalization in severe cases and typically involves systemic agents such as retinoids, cyclosporine, methotrexate, or biologics.

The biologics are of various kinds as some target the interleukin-36 (IL-36) pathway, while others target the IL-17 pathway. Prognoses of GPP depend on timely recognition and intervention [5]. Generalized pustular psoriasis not only deteriorates quality of life but also imposes considerable economic burdens on healthcare systems, and society as a whole [6].

Until recently, the empirical literature for GPP therapy lacked comparative interventional studies; for a long time, the use of therapies like methotrexate, isotretinoin, and cyclosporin—to name a few—were substantiated by evidence from case studies (i.e., case reports or case series) [7,8,9,10]. The rarity of GPP could partly be the reason there has been a paucity of interventional studies; for instance, when a diagnosis is rare, prospective studies may not be conducted as they would easily be underpowered, i.e., have a Type II error rate (which is often denoted as ‘β’) of over 20%. However, over the past lustrum, the evidence base has been enriched with publication of comparative studies, which, in turn, could facilitate the conduct of synthesis studies, such as the meta-analysis study by Chen et al. (2024) [7]. Furthermore, the use of validated metrics such as the Generalized Pustular Psoriasis Physician Global Assessment (GPPGA) and the Generalized Pustular Psoriasis Area and Severity Index (GPPASI) is evident in these comparative studies [11]. In 2022, the United States Food and Drug Administration (FDA) approved intravenous Spesolimab 900 milligrams (mg) for the treatment of GPP flares in adults [12]. Interleukin-17 inhibitors, including secukinumab, have also been used to treat GPP.

GPP prevalence varies geographically, ranging from 0.15 per 10,000 in Sweden to 5.0 per 10,000 in Brazil [13]. Sex distribution also varies, with female predominance reported in Sweden and Malaysia [14,15], but higher prevalence rates are observed across men in China [16].

GPP is an autoinflammatory disease driven by innate immune and IL-36 signaling, with variants in IL36RN, AP1S3, MPO, and related pathways implicated in the disease pathogenesis [1,4]. Flares may be triggered by infections, stress, pregnancy, and medications including corticosteroids and antimalarials.

GPP is primarily a clinical diagnosis characterized by widespread sterile pustules on erythematous non-acral skin, often with systemic symptoms [17,18]. The European Rare And Severe Psoriasis Expert Network (ERASPEN) and Japanese Dermatological Association (JDA) criteria emphasize pustulation, systemic involvement, and recurrence—all of which improves diagnostic consistency and comparability across studies [5,18,19]. Furthermore, a major diagnostic challenge with GPP is distinguishing it from acute generalized exanthematous pustulosis (AGEP), a condition that is usually drug-induced and resolves rapidly after stopping the triggering agent. Unlike AGEP, GPP typically follows a relapsing or persistent course and is often associated with a history of psoriasis [20].

The current study conducted a systematic review to narratively synthesize information regarding the impact of biologics on treatment of GPP. Secondarily, we conducted an exploratory network meta-analyses (NMAs) with the evidence we identified.

## 2. Methods

The conduct of our work was in accordance with the Preferred Reporting Items for Systematic Reviews and Meta-Analyses (PRISMA) guidelines for systematic reviews [21]. The protocol for our work was registered under Zenodo (https://doi.org/10.5281/zenodo.19498902) (Registration No. 19498902).

### 2.1. Search and Eligibility

Studies’ eligibility for our review was determined using the population, intervention, comparator, and outcome (PICO) framework. Outcome measures corresponded to Generalized Pustular Psoriasis Area and Severity Index (GPPASI) or Generalized Pustular Psoriasis Physician Global Assessment (GPPGA)—which are each validated outcome measures. Population corresponded to patients with GPP, regardless of their age, sex, ethnicity, or geographic location. Intervention corresponded to therapy with biologics—while comparators comprised other biologic therapies as well or inactive controls such as placebo or vehicle. Study design had to be comparative (i.e., at least 2 arms) and interventional; hence, single-arm studies and case reports were excluded. Only studies published in English were included in the review.

Initial systematic searches were conducted in PubMed and EMBASE in January 2026—and updated searches were done in April 2026. For PubMed, we used the following query: ((random*[Title/Abstract] OR compar*[Title/Abstract] OR effect*[Title/Abstract] OR effic*[Title/Abstract] OR trial*[Title/Abstract] OR impact*[Title/Abstract] OR treat*[Title/Abstract] OR therapy*[Title/Abstract]) AND ((biological therapy[MeSH Terms]) OR (biologic*[Title/Abstract]))) AND (“generalized pustular psoriasis”[Title/Abstract] OR “generalised pustular psoriasis”[Title/Abstract]). Records identified through the searches were imported to and managed with software that are used for management of systematic reviews, namely, Mendeley v1.69.3, Zotero 7.0.16 and Rayaan (accessed on 15 January 2026). Deduplication was achieved with the Evidence Review Accelerator (TERA) [22]. Two reviewers independently conducted screening of titles and abstracts (MAB and MT), followed by eligibility assessment of full-text articles. Data were extracted independently by two reviewers (MAB and MT) using a standardized form, capturing variables such as study and participant characteristics, intervention details, outcome measures, follow-up duration, and key findings. Any disagreements were resolved through discussion, with a third reviewer (AKG).

The risk of bias in included studies was assessed using Cochrane Collaboration tools. Non-randomized studies were evaluated with the ROBINS-I tool, while randomized studies used the Cochrane Risk of Bias (RoB) v2 tool. Assessments were made at the domain level to provide a nuanced understanding of methodological limitations.

### 2.2. Exploratory Network Meta-Analyses

Exploratory NMAs were conducted to determine the relative effectiveness of biologic agents investigated across the included studies. These analyses were considered ‘hypothesis-generating’ because a ‘confirmatory’ NMA was not deemed appropriate for conclusive inference-making.

## 3. Results

**Figure 1:**
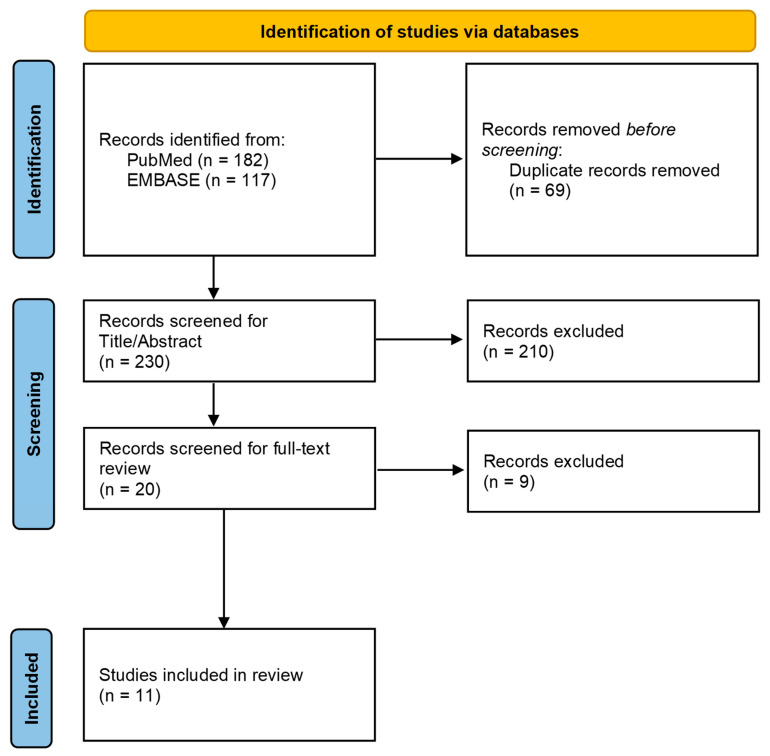
Identification of eligible studies.

We identified a total of 11 comparative interventional studies [23,24,25,26,27,28,29,30,31,32,33] that examined the impact of various therapies on GPP (Figure 1). These 11 studies comprise randomized and observational studies. The observational studies were of various designs: we identified subgroup analyses, post hoc analyses, prospective and retrospective cohort studies. Collectively, the 11 studies represent the recent evidence base of comparative studies on biologics for GPP (Table 1). A more expanded (i.e., detailed) version of Table 1 is available in the Supplementary Materials (Table S1). The sample size in most of these studies were limited due to the rarity of GPP; most studies had a small number of participants. Heterogeneity was also observed among the included studies, which could have implications for quantitative evidence syntheses like meta-analytic studies. Despite the heterogeneity, the included studies provide valuable insights into treatment effectiveness, patterns of response, and clinical outcomes across diverse settings.

### 3.1. Heterogeneity of Study Design, Study Populations and Treatment Regimens

As alluded to earlier, heterogeneity was evident across study designs, populations, and interventions. Observational studies are susceptible to residual confounding. Though observational, the retrospective cohorts, post hoc analyses, and subgroups are not equivalent study designs; in other words, they are methodologically distinct and not interchangeable. Furthermore, double counting of patients could feasibly occur across multiple post hoc studies especially if the parent trials are few and/or a disease of interest is rare. Therefore, findings from our exploratory NMAs are to be interpreted cautiously.

While randomized controlled trials such as Effisayil-1 and Effisayil-2 provided high-quality evidence using validated endpoints (e.g., GPPGA), a large proportion of our included studies were observational and were often characterized by smaller sample sizes and variable follow-up durations. Study populations varied in terms of geographic location and ethnicity (e.g., Chinese cohort vs. multinational patients). Baseline disease characteristics such as severity of GPP were inconsistently quantified as various severity indices were used across studies, including median GPPASI and population-specific measures such as the JDA severity index. There was also variability in prior treatment exposure: some studies required patients to be biologic-naïve while others included biologic-exposed patients—and some required patients be naïve for only certain biologics. Treatment regimens further contributed to heterogeneity, as they varied in dosage, route of administration, and treatment duration. The use of concomitant therapies was variably reported: some studies permitted them while others did not—thereby introducing additional confounding.

### 3.2. Broadly Convergent Trends: A Case for Future Quantitative Syntheses

Despite the methodological heterogeneity across the current GPP evidence base, directionally consistent patterns were identified, supporting the feasibility of formal quantitative syntheses like network meta-analyses in the near future. Across randomized controlled trials, subgroup analyses, and observational studies, biologic therapies consistently demonstrated clinically meaningful efficacy in both acute flare management and longer-term disease control.

IL-36 inhibition, particularly with spesolimab, demonstrated a rapid onset of action in a randomized controlled trial setting (i.e., high internal validity), with a substantial proportion of patients achieving pustular clearance within one week. These early clinical responses were complemented by longer-term findings, including reduced flare frequency, prolonged time to relapse, and sustained disease control over follow-up periods extending to 48 weeks and beyond. Notably, these effects were consistent across subgroup analyses. The directionally consistent findings are particularly notable given differences in route of administration, including both intravenous and subcutaneous delivery.

Other biologic classes, including IL-17 inhibitors (secukinumab, ixekizumab), IL-23 inhibitors (guselkumab), TNF inhibitors (adalimumab, certolizumab pegol, etanercept, infliximab), and IL-12/23 inhibitors (ustekinumab) also demonstrated favorable outcomes, particularly in real-world and cohort studies. High rates of clinical response were reported, including achievement of GPPASI 90 and GPPASI 100, sustained skin clearance, and reductions in recurrence over extended follow-up periods. These findings were observed across a range of patient populations—also including those with prior biologic exposure, highlighting the applicability of these therapies in routine clinical practice.

In addition to clinician-assessed outcomes, improvements in patient-reported outcomes were consistently reported across studies. These included reductions in pain, fatigue, and improvement in dermatology-related quality of life measures; improvements were often observed early in the treatment course and maintained over time. Emerging comparative data from observational studies also suggested potential differences in efficacy and recurrence profiles among biologic agents, although these findings remain exploratory and warrant further investigation.

Overall, the evidence demonstrates consistent and clinically meaningful benefits of biologic therapies in GPP. Across diverse study designs and patient populations, these therapies provide rapid symptom control, sustained disease improvement, and meaningful improvements in quality of life, supporting their role in both acute and long-term management strategies. Details of our exploratory NMAs are presented in the Supplementary Materials.

## 4. Discussion

This systematic review synthesizes the current comparative evidence on biologic therapies for GPP. Despite the limited sample sizes and methodological variability across studies, a consistent pattern of therapeutic benefit was observed. We found that findings from randomized controlled trials were supported by observational data—all of which support biologic therapies to be effective in both acute flare management and longer-term disease control. The directionally consistent findings across diverse study designs is particularly notable, as it suggests that the observed treatment effects are clinically meaningful, even in the context of heterogeneity. This consistency also rationalizes the conduct of more formal quantitative syntheses, such as network meta-analyses, as the evidence base continues to expand.

Mechanistically, the findings reinforce the central role of targeted immunomodulation in GPP. IL-36 inhibition, particularly with spesolimab, demonstrated rapid therapeutic relief and sustained efficacy, aligning with the known role of IL-36 signaling in disease pathogenesis. At the same time, the observed effectiveness of biologics targeting the IL-17, IL-23, TNF, and IL-12/23 pathways highlights that GPP is driven by multiple pathways. The efficacy of these agents across varied patient populations (including those with and without prior biologic exposure)—and thereby underscores their usefulness in real-world settings. Furthermore, consistent improvements in patient-reported outcomes emphasize that these therapies provide not only clinical disease control but also meaningful benefits in quality of life, which is a critical consideration in chronic inflammatory diseases such as GPP.

While the exploratory NMAs provide quantitative insights into the relative effectiveness of different biologic classes, its findings should be interpreted cautiously given the limited number of studies and small sample sizes. Nonetheless, the alignment between narrative and quantitative findings highlights promising directions for future research, including the need for larger, standardized studies and head-to-head comparisons to better define optimal therapeutic strategies in GPP.

## 5. Conclusions

In conclusion, this review highlights that despite the rarity of GPP and the resulting limitations in study size and methodological consistency of studies thereof, there is a directionally consistent body of evidence supporting the effectiveness of biologic therapies in both acute and long-term disease management. The findings across randomized and observational studies underscores the clinical relevance of these treatments, particularly those targeting key inflammatory pathways such as IL-36, IL-17, IL-23, TNF, and IL-12/23. While exploratory analyses provide preliminary comparative insights, further high-quality, standardized, and adequately powered studies are needed to refine treatment strategies and optimize patient outcomes.

## Figures and Tables

**Table 1:** Summary of the 11 studies that were included in our narrative syntheses.

Author	Objective	Interventions	Biologic Naïve	Other Therapy	N	Age	Sex	Findings (Summary)
Xu et al. 2026 [33]	To evaluate the effectiveness of various biologic therapies for GPP in real-world settings and to characterize the diversity of patient responses based on differing clinical characteristics.	TNF-alpha (etanercept, infliximab, adalimumab)	Yes	No	29	mean = 25.1 SD = 14.5	M = 18, F = 11	Patients receiving IL-36 inhibitors experienced the shortest hospital stays and demonstrated the most significant reductions in both GPPGA and GPPASI scores. Those with the highest baseline disease severity responded most favorably to IL-36 inhibitor therapy.
		IL-17A inhibitor (secukinumab or ixekizumab)	Yes	No	22	mean = 26.8, SD = 16.7	M = 12, F = 10	
		IL-36 receptor antagonist (spesolimab)	No	No	11	mean = 35.3, SD = 19.4	M = 6, F = 5	
Lu et al. 2024 [27]	A comparative analysis of the efficacy of adalimumab, secukinumab, and guselkumab in the treatment of generalized pustular psoriasis (GPP)	Adalimumab (subcutaneous)	Yes	Yes	15	mean = 51.7, SD = 15.6	M = 11, F = 4	According to the primary endpoint (GPPASI-75 at 12 weeks), Guselkumab demonstrated the highest efficacy, followed by Secukinumab and Adalimumab.
		Guselkumab (subcutaneous)	Yes	Yes	16	mean = 52, SD = 18.2	M = 12, F = 4	
		Secukinumab (subcutaneous)	Yes	Yes	19	mean = 46.4, SD = 13.4	M = 14, F = 5	
Navarini et al. 2023 [29]	Describe and quantify the impact of spesolimumab (IL-36R inhibitor) on GPP patients from the Effisayil-1 study insofar as patient-reported outcomes (PROs)	Spesolimab (intravenous)	Yes	No	35	mean = 43.2, SD = 12.1	M = 14, F = 21	Improvements in PROs were observed in the spesolimab arm, and patients who crossed over from the placebo arm to receive the active treatment also showed improvement. Overall, spesolimab demonstrated greater efficacy than placebo.
		Placebo			18	mean = 42.6, SD = 8.4	M = 3, F = 15	
Bachelez et al. 2021 Effisayil-1 NCT03782792 [23]	To investigate the efficacy of spesolimab as compared with placebo in patients with GPP flares	Spesolimab (intravenous)	Yes	No	35	mean = 43.2, SD = 12.1	M = 14, F = 21	At week 1, spesolimab was markedly more efficacious than placebo.
		Placebo			18	mean = 42.6, SD = 8.4	M = 3, F = 15	
Okubo et al. 2022 NCT03051217 [30]	Explore the efficacy of certolizumab pegol in patients with GPP and Erythrodermic Psoriasis (EP)	Certolizumab pegol	No	Yes	3	mean= 44.7, SD = 8.3	M = 1, F = 1	Improvements were observed in patients with GPP at both dosage levels, with no significant difference between them.
		Certolizumab pegol	No	Yes	4	mean = 51, SD = 14.6	M = 2, F = 2	
Burden et al. 2023 [24]	Subgroup analysis (of Effisayil 1 (NCT03782792) data) to determine the efficacy of spesolimab according to patient demographic clinical characteristics.	Spesolimab (intravenous)	Yes	No	35	mean = 43.2, SD = 12.1	M = 14, F = 21	Spesolimab was more effective than placebo across all demographic subgroups (e.g., sex, BMI, race).
		Placebo			18	mean = 42.6, SD = 8.4	M = 3 F = 15	
Morita et al. 2023 Effisayil-2 NCT03886246 [28]	To assess the efficacy of spesolimab in the prevention of GPP flares.	Spesolimab (subcutaneous)	No	Yes	31	mean = 38.9, SD = 16.5	M = 11, F = 20	Spesolimab was superior to placebo, with a dose–response relationship observed.
		Spesolimab (subcutaneous)	No	Yes	31	mean = 42.9, SD = 16.7	M = 11, F = 20	
		Spesolimab (subcutaneous)	No	Yes	30	mean = 40.2, SD = 16.4	M = 12, F = 18	
		Placebo (subcutaneous)			31	mean = 39.5, SD = 14.0	M = 13, F = 18	
Tsai et al. 2023 [32]	Explore the efficacy of spesolimab in subgroup of Chinese patients from the Effisayil-1 trial	Spesolimab (intravenous)			5	mean = 47.2 SD = 10	M = 2, F = 3	Findings in the Chinese subpopulation were consistent with those of the global population, as reported in Effisayil-1.
		Placebo			6	mean =42, SD = 11.1	M = 1, F = 5	
Hu et al. 2024 [26]	To explore the long-term effectiveness IL-17 inhibitors	Ixekizumanb (subcutaneous)			5	mean = 32.6, SD = 23.2	M = 3, F = 2	
		Secukinumab (subcutaneous)			13	mean = 22, SD = 12.8	M = 11, F = 2	
Ruan et al. 2024 [31]	To investigate how GPP patients with different genetic profiles’ response to ustekinumab and secukinumab	Ustekinumab (subcutaneous)	No	No	32	mean = 36, SD = 18.97	M = 15, F = 17	Efficacy was not dependent on mutation Bothe agents were effective, however secukinumab showed more promptness in effectiveness
		Secukinumab (subcutaneous)	No	No	33	mean = 22.92, SD = 21.07	M = 15, F = 18	
Gordon et al. 2025 [25]	To determine effect of spesolimab vs. placebo on sustaining improvement of GPP	Spesolimab (subcutaneous)	No	No	30	mean = 40.2, SD = 16.4	M = 12, F = 18	The effects of spesolimab were sustained much more than those who received placebo up till 48 weeks
		Placebo	No	No	31	mean = 39.5, SD = 14.0	M = 13, F = 18	

## Data Availability

The original contributions presented in this study are included in the article/Supplementary Materials. Further inquiries can be directed to the corresponding author.

## Supplementary Materials

The following supporting information can be downloaded at: https://www.mdpi.com/article/10.3390/medsci14020307/s1, Table S1: Summary of the 11 studies that were included in our narrative syntheses (expanded/full version); Appendix S1: Exploratory Network Meta-Analyses; Figure S1: Network plot for proportion of patients with generalized pustular psoriasis who attained maximum improvement (i.e., GPPASI-100 or GPPGA pustulation score of 0) at 12 weeks; Figure S2: Network plot for proportion of patients with generalized pustular psoriasis who attained maximum improvement (i.e., GPPASI-100 or GPPGA pustulation score of 0) at 48 weeks; Figure S3: A kilim plot of interventions’ surface under the cumulative ranking curve (SUCRA) values; Figure S4: League table for pairwise relative effects for proportion of patients with generalized pustular psoriasis who attained maximum improvement (i.e., GPPASI-100 or GPPGA pustulation score of 0) at 12 weeks; Figure S5: League table for pairwise relative effects for proportion of patients with generalized pustular psoriasis who attained maximum improvement (i.e., GPPASI-100 or GPPGA pustulation score of 0) at 48 weeks.
