# Supplementary material for: Monotherapy with Biologics for Generalized Pustular Psoriasis: A Systematic Review of Comparative Interventional Studies with an Exploratory Network Meta-Analysis
Source: Med Sci (Basel). 2026 Jun 11;14(2):307. doi: 10.3390/medsci14020307 (PMC13302897; doi:10.3390/medsci14020307)
Supplement: Supplementary file 1 [file medsci-14-00307-s001.zip › Supplementary Materials May 27 2026.pdf]

Table S1. Summary of the 11 studies that were included in our narrative syntheses (expanded/full version)

Author	Diagnostic criteria	Genetic testing	Objective	Study design and data sources	Outcome measures	Interventions	Biologic naïve	Other therapy	N	Age	Sex	Findings (summary)
Xu et al 2026 [33]	European Rare and Severe Psoriasis Expert Network (ERASPEN) criteria for GPP<br><br>GPPGA score ≥ 2	No	To evaluate the effectiveness of various biologic therapies for GPP in real-world settings and to characterize the diversity of patient responses based on differing clinical characteristics.	Retrospective Cohort<br><br>Laboratory data from clinical records<br><br>Country: China	**Primary follow up period:** Not defined<br><br>**Primary outcome** Not defined<br><br>**Other outcome(s):** -Length of hospital stay -Change in GPPGA -Change in GPPASI	TNF-alpha (etanercept, infliximab, adalimumab)	Yes	No	29	mean = 25.1<br><br>SD = 14.5	M= 18, F= 11	Patients receiving IL-36 inhibitors experienced the shortest hospital stays and demonstrated the most significant reductions in both GPPGA and GPPASI scores. Those with the highest baseline disease severity responded most favorably to IL-36 inhibitor therapy.
						IL-17A inhibitor (secukinumab or ixekizumab))	Yes	No	22	mean = 26.8,<br><br>SD = 16.7	M= 12, F= 10	
						IL-36 receptor antagonist (spesolimab)	No	No	11	mean = 35.3,<br><br>SD = 19.4	M= 6, F = 5	

Author	Diagnostic criteria	Genetic testing	Objective	Study design and data sources	Outcome measures	Interventions	Biologic naïve	Other therapy	N	Age	Sex	Findings (summary)
Lu et al 2024 [27]	No details	No	A comparative analysis of the efficacy of adalimumab, secukinumab, and guselkumab in the treatment of generalized pustular psoriasis (GPP)	Prospective Cohort<br><br>Patients visiting dermatology clinics at a tertiary care center<br><br>Country: China	**Primary follow up period**: 12 weeks<br><br>**Primary Outcome:** GPPASI-75 at 12 weeks<br><br>**Other Outcome(s):**<br>-change in GPPASI at 0,4,8,12 weeks<br>-GPPASI-75 at 0,4 and 8 weeks<br>-GPPASI-90 at 0,4,5,12 weeks<br>-GPPGA 0/1 at 0,4,8 and 12 weeks<br>-Change in BSA at 0,4,8 and 12 weeks<br>-Time to pustule clearance<br>-Length of hospital stay	Adalimumab (subcutaneous)<br><br>80 mg initially, followed one week later by 40 mg every 2 weeks.	Yes	Yes	15	mean = 51.7,<br><br>SD = 15.6	M=11, F= 4	According to the primary endpoint (GPPASI-75 at 12 weeks), Guselkumab demonstrated the highest efficacy, followed by Secukinumab and Adalimumab.
						Guselkumab (subctaneous)<br><br>100 mg at week 0,4 and then every 8 weeks	Yes	Yes	16	mean = 52, SD = 18.2	M= 12, F= 4	
						Secukinumab (subcutaneous)<br><br>300 mg at week 0,1,2,3,4 then every 4 weeks	Yes	Yes	19	mean = 46.4, SD = 13.4	M= 14, F= 5	

Author	Diagnostic criteria	Genetic testing	Objective	Study design and data sources	Outcome measures	Interventions	Biologic naïve	Other therapy	N	Age	Sex	Findings (summary)
Navarini et al 2023 [29]	-GPPGA score ≥ 3<br>-GPPGA pustulation subscore ≥ 2<br>- ≥5% of body surface area with erythema and the presence of pustules	Yes	Describe and quantify the impact of spesolimumab (IL-36R inhibitor) on GPP patients from the Effisayil -1 study inso far as patient reported outcomes (PROs)	Post hoc analysis of data from the Effisayil-1 study	**Primary follow up period:**<br>12 weeks<br><br>**Primary Outcomes:**<br>-pain VAS at baseline, Day 8, Weeks 2 – 4, 8 and 12,<br>-FACIT-Fatigue at baseline, Day 8, Weeks 2 – 4, 8 and 12,<br>-DLQI at baseline, Day 8, Weeks 2 – 4, 8 and 12,<br>-PSS at baseline, Day 8, Weeks 2 – 4, 8 and 12	Spesolimab (intravenous)<br><br>900 mg — single dose<br>optional second dose (open label) at Day 8 depending on treatment progression	Yes	No	35	mean = 43.2,<br><br>SD = 12.1	M=, 14<br>F= 21	Improvements in PROs were observed in the spesolimab arm, and patients who crossed over from the placebo arm to receive the active treatment also showed improvement. Overall, spesolimab demonstrated greater efficacy than placebo.
						Placebo			18	mean = 42.6,<br>SD = 8.4	M=3,<br>F =15	
Bachelez et al 2021 Effisayil-1 NCT03782792 [23]	1. ERASPEN<br>2. GPPGA score ≥ 3<br>3. ≥5% of body surface area with erythema	Yes	To investigate the efficacy of spesolimab as compared with placebo in	Randomized Controlled Trial<br>Phase II<br>Crossover<br>Country: Multiple countries	**Primary follow up period:**<br>1 week<br><br>**Primary outcome:**<br>GPPGA pustulation	Spesolimab (intravenous)<br><br>900 mg — single dose<br>optional second dose (open label) at Day 8 depending on	Yes	No	35	mean = 43.2,<br>SD = 12.1	M=14,<br>F = 21	At week 1, spesolimab was markedly more efficacious than placebo.

Author	Diagnostic criteria	Genetic testing	Objective	Study design and data sources	Outcome measures	Interventions	Biologic naïve	Other therapy	N	Age	Sex	Findings (summary)
	and the presence of pustules		patients with GPP flares		subscore 0 at week 1<br><br>**Other Outcome(s)**<br>-GPPGA pustulation subscore 0 or 1 at week 1<br>-GPPASI 75 at week 4	treatment progression						
						Placebo			18	mean = 42.6, SD = 8.4	M=3, F =15	
Okubo et al 2022 NCT03051217 [30]	JDA criteria	No	Explore the efficacy of certolizumab pegol in patients with GPP and Erythrodermic Psoriasis (EP)	Randomized Controlled Trial Blinded<br><br>Phase II/III<br><br>Country: Japan	**Primary follow up period:**<br>52 weeks<br><br>**Primary outcomes:**<br>-Clinical Global Impression of Improvement response<br>-DLQI<br>-INRS<br>-GIS<br>-JDA Severity Index	Certolizumab pegol<br><br>400 mg — every 2 weeks for 52 weeks	No	Yes	3	mean = 44.7, SD = 8.3	M =1, F = 1	Improvements were observed in patients with GPP at both dosage levels, with no significant difference between them.
						Certolizumab pegol<br><br>200 mg — every 2 weeks for 52 weeks	No	Yes	4	mean = 51, SD = 14.6	M =2, F=2	

Author	Diagnostic criteria	Genetic testing	Objective	Study design and data sources	Outcome measures	Interventions	Biologic naïve	Other therapy	N	Age	Sex	Findings (summary)
Burden et al 2023 [24]	ERASPEN	Yes	Subgroup analysis (of Effisayil 1 (NCT03782792) data) to determine the efficacy of spesolimab according to patient demographic clinical characteristics	Subgroup analysis of NCT03782792 included groups with at least 5 patients and a minimum of 2 categories. Subgroups were defined by sex (male, female); BMI (<25 kg/m², 25 to <30 kg/m², ≥30 kg/m²); plaque psoriasis (yes, no); IL36 mutation status (yes, no); GPPGA total score; GPPGA pustulation subscore; median GPPASI score; JDA GPP severity index; and use of medication prior to randomization.	Primary follow up period: 1 week Primary outcome: GPPGA pustulation subscore 0 at week 1	Spesolimab (intravenous) 900 mg	Yes	No	35	mean = 43.2, SD = 12.1	M=14, F = 21	Spesolimab was more effective than placebo across all demographic subgroups (e.g., sex, BMI, race).
						Placebo			18	mean = 42.6, SD = 8.4	M = 3 F =15	

Author	Diagnostic criteria	Genetic testing	Objective	Study design and data sources	Outcome measures	Interventions	Biologic naïve	Other therapy	N	Age	Sex	Findings (summary)
Morita et al 2023 Effisayil-2 NCT03886246 [28]	ERASPEN GPPGA = 0 or 1	Yes	To assess the efficacy of spesolimab in the prevention of GPP flares.	Randomized controlled Trias<br><br>Phase IIb<br><br>Country: Multiple countries	Follow up: 48 weeks Primary: Time to first GPP flare by week 48 Secondary: 1. occurrence of at one GPP flare by week 48	Spesolimab (subcutanous)<br><br>300 mg loading dose, then 150 mg maintance dose every 12 weeks till week 44	No	Yes	31	mean = 38.9, SD = 16.5	M= 11, F = 20	Spesolimab was superior to placebo, with a dose–response relationship observed.
						Spesolimab (subcutaneous)<br><br>600 mg loading dose, then 300 mg till week 44 maintaince dose every 12 weeks	No	Yes	31	mean = 42.9, SD = 16.7	M =11, F =20	
						Spesolimab (sbcutaneous)<br><br>600 mg loading dose, then 300 mg maintaince dose every 4 weeks till week 44	No	Yes	30	mean = 40.2, SD = 16.4	M = 12, F = 18	
						Placebo (subcutneus)			31	mean = 39.5, SD = 14.0	M = 13, F = 18	

Author	Diagnostic criteria	Genetic testing	Objective	Study design and data sources	Outcome measures	Interventions	Biologic naïve	Other therapy	N	Age	Sex	Findings (summary)
Tsai et al 2023 [32]	ERASPEN	Yes	Explore the efficacy of spesolimab in subgroup of Chinese patients from the Effisayil - 1 trial	Subgroup analyses of Effisayil - 1 data	**Primary follow up:** 1 week **Primary outcome:** GPPGA of 0 at week 1 **Other outcome(s):** GPPGA o or 1 at week 1	Spesolimab (intravenous) 900 mg			5	mean = 47.2 SD = 10	M =2, F = 3	Findings in the Chinese subpopulation were consistent with those of the global population, as reported in Effisayil-1.
						Placebo			6	mean =42, SD = 11.1	M =1, F = 5	
Hu et al 2024 [26]	ERASPEN	Yes	To explore the long-term effectiveness IL-17 inhibitors	Retrospective cohort Hospital data	**Primary follow up:** 96 weeks **Primary outcme:** GPPASI 90 or 100 at week 96 **Other outcome(s):** change in GPPASI at 96 weeks DLQI 0/1 at 96 weeks,	Ixekizumanb(subcutaneous)			5	mean = 32.6, SD = 23.2	M=3, F =2	
						Secukinumab (subcutenaous)			13	mean = 22, SD = 12.8	M=11, F =2	

Author	Diagnostic criteria	Genetic testing	Objective	Study design and data sources	Outcome measures	Interventions	Biologic naïve	Other therapy	N	Age	Sex	Findings (summary)
Ruan et al 2024 [31]	JDA criteria	Yes	To investigate how GPP patients with dofferent genetic profiles respons to ustekinumab and secukinumab	Retrospective cohort, clinical and laboratory from clinical records<br>Country: China	**Primary follow up period:** Not defined<br>**Primary outcome for effectiveness:** Not defined<br>**outcomes:** *changes in body temperature and laboratory indices, improvement of skin lesions, and changes in quality of life*	Ustekinumab (subcustaneous)<br><br>Adults:<br>45 mg for adults weighing 100 kg or less<br>90 mg for adults weighing over 100 kg<br><br>Pediatrics:<br>The ustekinumab dosage for pediatric patients was 0.75 mg/kg for patients weighing < 60 kg; 45 mg for pediatric patients weighing 60 kg to 100 kg; and 90 mg for pediatric patients weighing > 100 kg<br><br>Week 0,1,2,3,4 and every 4 weeks till week 48	No	No	32	mean = 36, SD = 18.97	M = 15, F = 17	Efficiacy was not depednet on mutation Bothe agents were effective, however secuu showd more promptness in effectiveness

Author	Diagnostic criteria	Genetic testing	Objective	Study design and data sources	Outcome measures	Interventions	Biologic naïve	Other therapy	N	Age	Sex	Findings (summary)
						Secukinumab (subcutaneous)<br><br>Adults:<br>300 mg secukinumab<br><br>Pediatric patients received a dose based on their weight category (<50 kg, ≥50 kg): patients weighing < 50 kg received<br>75 mg, those weighing ≥50 kg received 150 mg	No	No	33	mean = 22.92, SD = 21.07	M=15, F = 18	
Gordon et al 2025 [25]	ERASPEN	Yes	To determine effect of spesolimab vs placebo on sustaining improvement of GPP	Post hoc analysis of data from the Effisayil 2 trial	**Primary follow up period:**<br>48 weeks<br>**Primary outcome for effectiveness:**<br>Proportion of GPP patients who sustained imporvement by week 48	Spesolimab (subcutaneous)<br><br>600 mg	No	No	30	mean = 40.2, SD = 16.4	M =12, F = 18	The effects of spesolimab were sustained much more than thos who received placebo up till 48 weeks
		Yes				Placebo	No	No	31	mean = 39.5, SD = 14.0	M = 13, F = 18	

Exploratory Network Meta-Analyses:

In addition to the narrative synthesis, an exploratory network meta-analysis (NMA) was conducted to provide a preliminary quantitative assessment of the relative effectiveness of biologic therapies for GPP given the current evidence base. This analysis followed PRISMA guidance for network meta-analysis and employed a Bayesian fixed-effects model to estimate relative treatment effects and efficacy ranks. Given the limited number of eligible comparative studies and the heterogeneity in study designs, populations, and interventions, the NMA was undertaken as a hypothesis-generating exercise rather than a definitive comparative evaluation. To enable synthesis across studies, "maximum improvement" was defined as achievement of either GPPASI-100 or a GPPGA pustulation score of 0. The two are clinically comparable measures of disease clearance—albeit, the amalgamation thereof introduces construct heterogeneity.

The NMA incorporated data from 4 of the 11 included studies and evaluated multiple biologic agents, including IL-17 inhibitors, IL-23 inhibitors, IL-12/23 inhibitors, IL-36 inhibitors, and placebo. Two outcome networks were constructed corresponding to 12-week and 48-week timepoints. Although the geometry of the network enabled indirect comparisons across interventions, it precluded formal inconsistency testing. Results of the NMAs, including network plots, league tables, and ranking visualizations, are presented in the **Supplementary Materials** (Figures S1–S5).

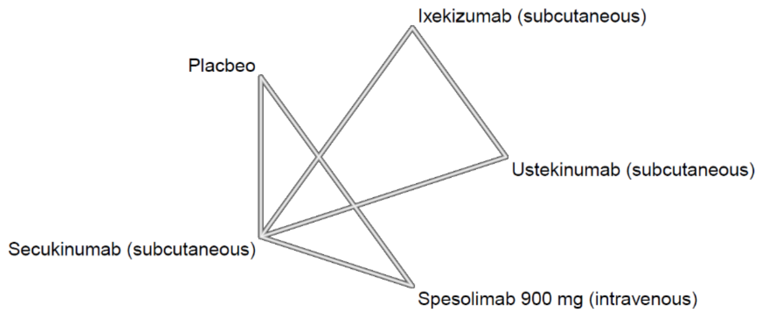

Figure S1. Network plot for proportion of patients with generalized pustular psoriasis who attained maximum improvement (i.e., GPPASI-100 or GPPGA pustulation score of 0) at 12 weeks.

Abbreviations

GPPASI: Generalized Pustular Psoriasis Area and Severity Index

GPPGA: Generalized Pustular Psoriasis Physician Global Assessment

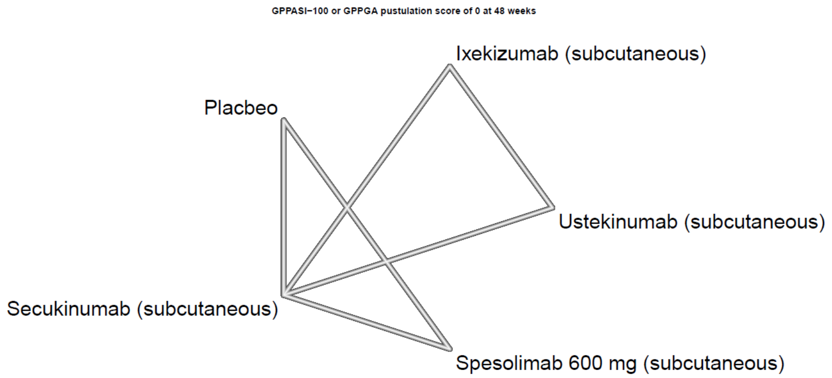

Figure S2 Network plot for proportion of patients with generalized pustular psoriasis who attained maximum improvement (i.e., GPPASI-100 or GPPGA pustulation score of 0) at 48 weeks.

Abbreviations

GPPASI: Generalized Pustular Psoriasis Area and Severity Index

GPPGA: Generalized Pustular Psoriasis Physician Global Assessment

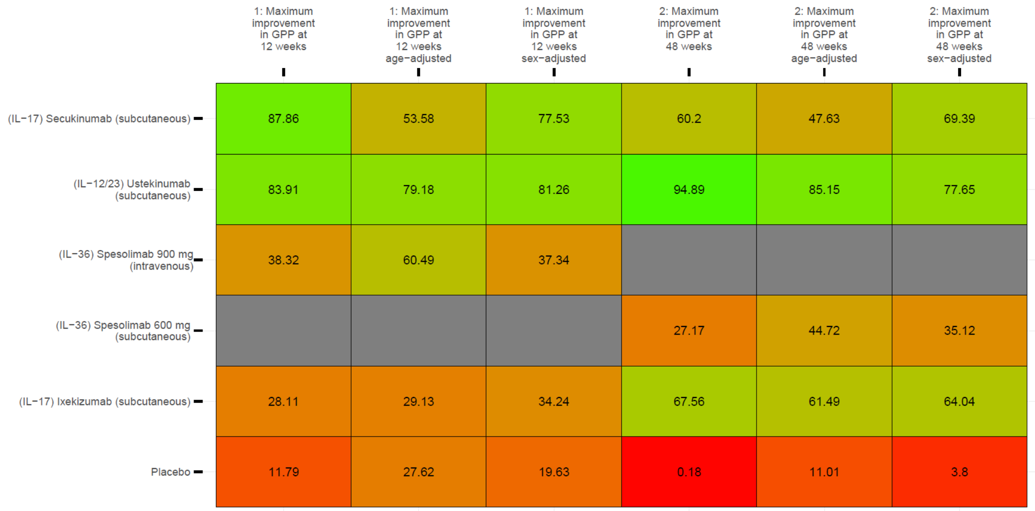

Figure S3. A kilim plot of interventions' surface under the cumulative ranking curve (SUCRA) values. Presented herein are comparators' relative effectiveness for the 2 outcomes under the base and sensitivity analyses. The numeric values in the cells represent each intervention's SUCRA. The horizontal axis represents the outcome, while the vertical axis corresponds to the distinct nodes (i.e., the comparators). A kilim plot is a tool that aims to intuitively—and easily—make comparators' relative effectiveness visually comparable by use of a colour gradient. In this kilim plot, greener cells indicate higher effectiveness, while redder cells indicate lower effectiveness. On the vertical axis, each comparator is prefixed by the type of agent it is; furthermore, IL refers to interleukin inhibitor.

**(IL-17) Secukinumab (Subcutaneous)**				**Evidence of treatment effect**
0.08 (-1.35, 1.63)	**(IL-12/23) Ustekinumab (Subcutaneous)**			**U.S. FDA Approved for GPP**
**2.1 (0.09, 4.36)**	2.02 (-0.55, 4.69)	**(IL-36) Spesolimab 900 mg (Intravenous)**		
**2.61 (0.12, 5.84)**	**2.53 (0.16, 5.69)**	0.51 (-2.82, 4.36)	**(IL-17) Ixekizumab (Subcutaneous)**	
**3.61 (1.35, 6.7)**	**3.53 (0.73, 6.9)**	1.51 (-1.63, 5.13)	1 (-2.82, 5.15)	**Placebo**

Figure S4. League table for pairwise relative effects for proportion of patients with generalized pustular psoriasis who attained maximum improvement (i.e., GPPASI-100 or GPPGA pustulation score of 0) at 12 weeks.

Abbreviations

GPPASI: Generalized Pustular Psoriasis Area and Severity Index

GPPGA: Generalized Pustular Psoriasis Physician Global Assessment

Evidence of treatment effect

(IL-12/23) Ustekinumab (Subcutaneous)				
1.01 (-1.29, 3.21)	(IL-17) Ixekizumab (Subcutaneous)			
1.32 (-0.14, 2.81)	0.31 (-1.94, 2.81)	(IL-17) Secukinumab (Subcutaneous)		
**2.88 (0.83, 4.92)**	1.87 (-0.74, 4.71)	**1.55 (0.24, 2.97)**	(IL-36) Spesolimab 600 mg (Subcutaneous)	
**4.54 (2.49, 6.6)**	**3.53 (0.93, 6.35)**	**3.22 (1.87, 4.72)**	**1.66 (0.57, 2.79)**	Placebo

Figure S5. League table for pairwise relative effects for proportion of patients with generalized pustular psoriasis who attained maximum improvement (i.e., GPPASI-100 or GPPGA pustulation score of 0) at 48 weeks.

Abbreviations

GPPASI: Generalized Pustular Psoriasis Area and Severity Index

GPPGA: Generalized Pustular Psoriasis Physician Global Assessment
